# Eating disorders and oral health: a scoping review on the role of dietitians

**DOI:** 10.1186/s40337-020-00325-0

**Published:** 2020-10-13

**Authors:** Tiffany Patterson-Norrie, Lucie Ramjan, Mariana S. Sousa, Lindy Sank, Ajesh George

**Affiliations:** 1 Centre for Oral Health Outcomes & Research Translation (COHORT), School of Nursing and Midwifery , Western Sydney University/South Western Sydney Local Health District/ Ingham Institute for Applied Medical Research, Liverpool BC, Locked Bag 7103, Sydney, NSW 1871 Australia; 2 School of Nursing and Midwifery, Western Sydney University, Centre for Oral Health Outcomes & Research Translation (COHORT), Sydney, Australia; 3 IMPACCT, Faculty of Health, University of Technology Sydney, Sydney, Australia; 4 Sydney Dental Hospital, Oral Health Services, SLHD, Sydney, Australia; 5 Centre for Oral Health Outcomes & Research Translation (COHORT), Western Sydney University/South Western Sydney Local Health District/University of Sydney/ Ingham Institute for Applied Medical Research, Sydney, Australia

**Keywords:** Dietitians, Early intervention, Oral health, Feeding and eating disorders, Health personnel

## Abstract

**Background:**

Compromised nutritional intake due to eating disorder related behaviors, such as binge eating and purging, can lead to multi-system medical complications, including an irreversible impact on oral health. However, dental anxiety, fear or embarrassment may hinder individuals with an eating disorder from seeking assistance for their oral health concerns. As key health professionals in eating disorder treatment, dietitians are well positioned to provide basic dental screening, however, their capacity to perform this role in practice has not been established. The aim of this review was to identify current evidence on the role of dietitians in promoting oral health among individuals with eating disorders.

**Methods:**

A comprehensive search of eight electronic databases and the grey literature was conducted to address the following three focus areas: 1) guidelines and recommendations on the role of dietitians in oral health 2) knowledge, attitudes and practices of dietitians regarding oral health promotion and; 3) current models of oral health care and resources for dietitians.

**Results:**

Twelve articles were included. The review indicated that current national and international position statements encourage dietitians to conduct basic oral health screening and promote oral health in high risk populations, such as those with an eating disorder. However, no evidence was found to indicate dietitians performed oral health screening or education in populations with an eating disorder. In other population settings, dietitians were found to play a role in oral health promotion, however, were noted to have mixed knowledge on oral health risk factors, prevention and treatment and generally were not providing referrals. Some oral health promotion resources existed for dietitians working in pediatric, HIV and geriatric clinical areas however no resources were identified for dietitians working in eating disorder settings.

**Conclusion:**

Despite current evidence showing that dietitians can play a role in oral health care, no models of care exist where dietitians promote oral health among individuals with an eating disorder. There are also no training resources and screening tools for dietitians in this area. Further research is required to develop this model of care and assess its feasibility and acceptability.

## Plain English summary

Eating disorder related behaviors including binge eating and purging are known to lead to significant medical and dental complications. Barriers including dental anxiety or embarrassment may hinder individuals with an eating disorder from seeking assistance for their oral health concerns. Dietitians form part of the primary care team for eating disorders and therefore are well positioned to provide basic dental screening and education, however, their capacity to perform this role in practice has not been established. A review of the literature was conducted and focused on guidelines for oral health promotion, dietitian knowledge, attitudes and practices towards oral health promotion, and the availability of resources in this area. Recommendations that supported the role of the dietitian in oral health promotion were identified. Additionally, dietitians were found to be aware of the importance of oral health, however were not providing referrals. Overall, there was limited evidence of adequate oral health resources to assist dietitians. Despite the limited evidence, it highlights their capability to provide pre-emptive oral health promotion in other clinical settings. Further research is needed to explore how to support dietitians to promote oral health among populations with an ED.

## Background

The prevalence, incidence and magnitude of eating disorders (ED) is increasing worldwide [1–3]. Around 1.2 million people in the United Kingdom [4], and approximately 30 million individuals in the United States are thought to currently have an eating disorder [5–7]. In Australia, ED are estimated to affect 4–9% of the population [8–10] and are the second leading cause of mental disorder disability among females [5, 8, 10, 11]. Eating disorders affect an individual’s social and functional roles, and increase overall risk of morbidity and mortality [5, 12]. From an economic standpoint, ED can also place significant financial strain on the individual and health system with the total burden of disease in Australia estimated to be $52.6 billion per year [13], with the impact on productivity reaching $15 billion [10].

Compromised nutritional intake as a result of restrictive or obsessive dieting and purging.

behaviors among people with ED can lead to multi-system medical complications such as bradycardia, electrolyte imbalance and renal failure [1, 12, 14]. Somewhat less well known, these behaviors can also have an irreversible impact on oral health [15, 16]. The results from two systematic reviews and meta-analyses confirmed an association between tooth erosion, poor oral health and ED. Individuals with an ED were five times more likely to have tooth erosion and overall higher decay, regardless of ED subtype [17, 18]. Furthermore, ED related dental complications can perpetuate body dissatisfaction leading to a decline in self-esteem, quality of life and psychosocial functioning [19–21]. When combined with the psychological and emotional stress of managing an ED, the impact of having an oral health complication can exacerbate ED signs and symptoms such as limited oral intake or food avoidance and inhibit treatment goals [20].

It is well known that good oral health is integral to general health, yet there are a number of barriers that may deter individuals from prioritizing their oral health care practices and seeking treatment for their oral health concerns. In vulnerable low income populations, individuals reported oral health behaviors such as living with chronic dental issues including dental pain or decaying teeth without seeking intervention, and accessing dental services only when dental concerns became unbearable [22, 23]. Significant relationships between poorer self-reported oral health outcomes, lower socioeconomic status and mental health vulnerabilities were also noted [22–24]. Specifically for individuals with a mental health condition, barriers to maintaining oral health included a reduced awareness of the presence/risk of oral health problems, the affect of medications such as antidepressants resulting in manifestations such as dry mouth, lower self-esteem and body image, poor diet and fear and distrust of dental providers [24–26]. Although individuals with ED were generally found to be concerned about their teeth especially the long term impact of dental issues such as enamel erosion [27, 28], their perceived barriers for not seeking dental intervention included reduced energy levels, anxiety, uncertainty about oral hygiene and distrust of dental providers [27, 28]. If left untreated, oral health complications can impede dietary intervention and ongoing ED treatment due to dental pain or discomfort [29–31].

Given the risk of dental problems among individuals with ED and their risk of poorer oral health outcomes, it is important to consider the promotion of oral health in this population. Previous research has supported the role of non-dental health professionals in raising awareness of dental problems and performing screening assessments in vulnerable or at-risk populations [30, 31]. Dietitians form an integral part of the multidisciplinary primary care team often working towards the stabilization of acutely unwell patients and helping to safely assist the client towards re-nourishment, relapse prevention and recovery [32–34]; this places them in a unique position to promote oral health in an ED clinical setting. However, to date, the potential role of the dietitian in promoting oral health among people with ED has received little attention and has not been clearly defined.

The aim of this scoping review was to identify current evidence supporting the role and scope of dietitians in this area. Specifically, this review was guided by the following focus areas:

- Guidelines and recommendations on the role of dietitians in oral health
- Knowledge, attitudes and practices (KAP) of dietitians in oral health promotion
- Current models of oral health care and resources for dietitians

### Terminology

#### Dietitian/ registered dietitian

A dietitian has tertiary qualifications in nutrition and dietetics which specifically involves the study of medical nutrition therapy, dietary counselling and food service management in addition to qualifications of a nutritionist. To obtain these qualifications, a dietitian must have “undertaken a course of study that included substantial theory and supervised and assessed clinical practice” [35].

#### Nutritionist

A nutritionist is a tertiary qualified professional who has the expertise to provide a range of nutrition services related to public health nutrition and community health [35].

#### Non-dental professional

An individual that is not recognized as a dentist, dental hygienist, dental therapist or other qualified oral health professional. Therefore, a non-dental professional can include, but is not limited to, nutritionists, dietitians, medical doctors, nurses, midwives and other allied health professionals.

## Methods

### Design

Utilizing the framework as described by Arksey and O’Malley, a scoping review was undertaken to investigate and summarize the nature and breadth of the role and scope of dietitians in providing oral health promotion (OHP) for individuals with ED, as well as to identify current gaps in the literature [36]. A scoping review was chosen as it allows for the researcher to follow an iterative search approach enabling the focus areas to be remodeled and re-defined especially given the paucity of research on this topic, which would increase the complexity of conducting a review.

### Search strategy

A preliminary search was undertaken by author TPN using Google Scholar to identify keywords based on published abstracts and articles. A total of eight databases were then searched including: MEDLINE (Ovid), Embase, EBSCO, PubMed, Cochrane, SCOPUS, Web of Science and ProQuest. Search strategies were enabled by Boolean operators (AND, OR, NOT), Truncations e.g. (diet*), medical subject headings (MESH) and descriptive key-terms where appropriate. In addition, a grey literature search was conducted to source government or non-government related material to assist in answering the aims of this review. Keywords used included: dietitian, oral health assessment, oral health screening, eating disorder, bulimia nervosa, anorexia nervosa, guidelines, training, resources, knowledge, attitudes, perceptions, practices, behaviors.

### Inclusion and exclusion criteria

All studies published up until October 2019 that addressed at least one of the focus areas were included in this review. There were no restrictions placed on database searches in terms of year of publication, study design or study quality for all focus areas. Only articles published in English were eligible for inclusion.

Articles and resources found on the world wide web were eligible for inclusion if the sources originated from a reputable research foundation, network, association, organization or government source. There was no restriction placed on year of publication for grey literature. Depending on the country of registration, dietitians can be identified by different titles such as dietitian, registered dietitian, registered dietitian nutritionist and so on. Therefore, due to the paucity of research in this area, studies that included dietitians or nutritionists were included in this study.

### Data screening, selection and extraction

**Fig. 1:**
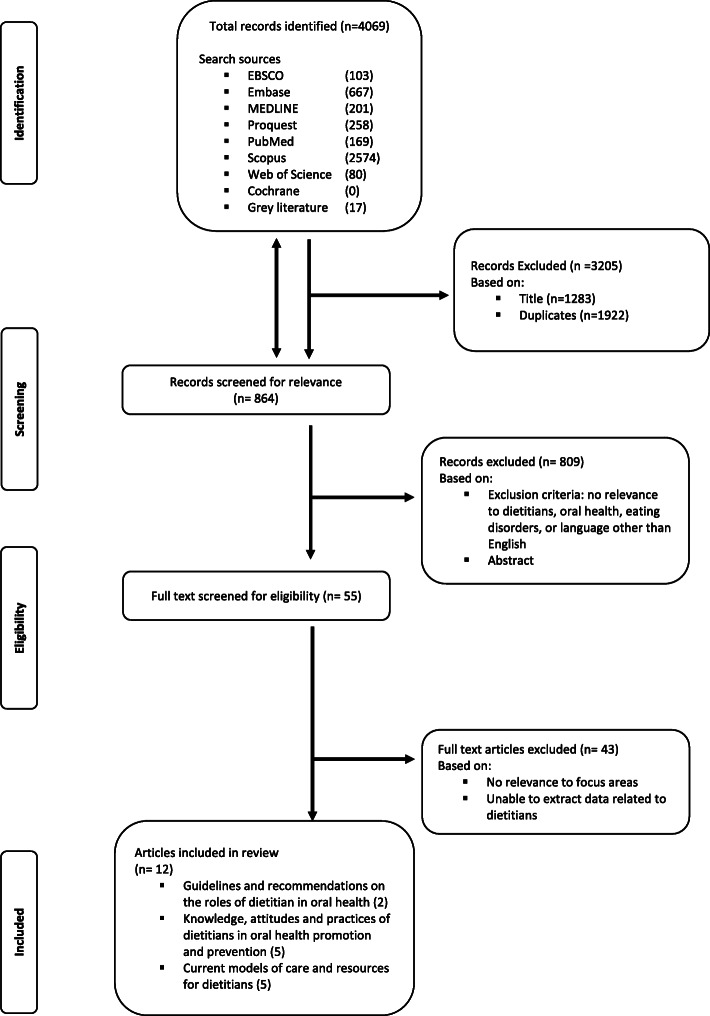
PRISMA flow chart indicating search strategy

The screening and selection were carried out by two authors (TPN, MSS). The process can be viewed in Fig. 1. Articles that met at least one of the inclusion requirements were included. All included articles were then categorized into the three focus areas. Initial results indicated that more expansive searching was required in all focus areas based on the meagre results acquired. The focus areas were revised from specifically investigating the ED population to other settings where dietitians were playing a role in oral health promotion. Data were extracted by one reviewer (TPN) and verified by three reviewers (MSS, LR, AG). Information extracted from the articles included: location of study, article type, study aims, study design, details of the study including participant demographics, study findings and conclusions.

## Results

A search of the literature yielded a total of 4069 records. Of these, 3205 were excluded based on title (*n* = 1283) and duplicates (*n* = 1922), resulting in 864 abstracts which were screened for relevance. From this, 55 articles were identified for full text review and a total of 12 articles were included in this scoping review. The articles were categorized under the following focus areas: i) Guidelines and recommendations on the role of dietitians in oral health (*n* = 2) ii) Knowledge, attitudes and practices (KAP) of dietitians in oral health promotion (*n* = 5) and; iii) current models of oral health care and resources for dietitians (*n* = 5) (Table 1). Literature and resources identified in the focus areas originated from Australia (*n* = 3 [38, 46, 49]), United States (*n* = 8 [37, 39–44, 48]), and Israel (*n* = 1 [45]).

**Table 1:** Included articles that identify available guidelines for dietitians, KAP of dietitians regarding oral health promotion and current models of oral health care and resources for dietitians

Author (Year)/ Location	Article Type	Aims	Study Design	Details of Study	Conclusion	Focus area
Decker, R.T. (2013) USA [37]	Position statement	To provide the position of The Academy of Nutrition and Dietetics regarding oral health and nutrition as well as describe the roles and responsibilities of dietetics practitioners in oral health education, practice and research.	N/A	Position statement on the role of the dietitians in oral health promotion	• The Academy supports the collaboration and integration of oral health with nutrition services, education and research. • Skills of screening oral health and making referrals is essential for dietitians. • Supports education that reinforces and illustrates the role of nutrition in oral health. • Collaboration between oral health practitioners and dietitians is recommended. • Eating disorders is highlighted as an at-risk group and some oral health manifestations typical to this population, such as xerostomia are specified. However, no specific information exists to detail the support dietitians should provide to this population. • A general table is provided where dietitians can identify oral related manifestations of nutrition deficits.	Guidelines and recommendations
Dietitians Australia and Dental Health Services Victoria (2015) Australia [38]	Joint Position statement	To provide evidence based oral health information to dietitians; to guide how oral health can be incorporated into the various roles of dietitians and to provide a framework for workforce capacity building workforce.	N/A	Joint position statement on the role of the dietitian in oral health promotion	• There is consensus that the dietitian’s role, dependent on setting, should: incorporate oral health screening, especially in priority at risk groups; recognize risk factors; provide nutritional management and/or provide guidance/ referral to oral health professionals; develop and deliver health promotion and education messages. • Specific oral health advice for dietitians is provided for medically compromised/special needs patients including individuals with an eating disorder. • Specific oral health advice for dietitians is provided for medically compromised/special needs patients including individuals with an eating disorder. Key advice includes encouraging good oral hygiene and providing advice on oral care after vomiting.	Guidelines and recommendations
Faine, M.P. (1995) USA [39]	Peer reviewed journal article	Assess the knowledge of the role of diet in dental caries aetiology in nutritionists and dental hygienists.	Cross-sectional	136 registered dietitians and 37 registered dental hygienists	• Awareness varied across different topics related to caries-preventative measures. While 95% of nutritionists recognised fluoride uptake into developing teeth makes dental enamel more resistant to dental caries, only 16% understood that fluoride may hinder bacterial activity. • Most nutritionists (53%) demonstrated awareness of the infectious nature of dental caries but only 38% recognised mothers can transmit bacteria to their children. • Nearly all nutritionists correctly identified high vs. low cariogenic snack foods. Nearly all (97%) of nutritionists were aware of and discussed childhood caries with clients. • Two thirds of nutritionists surveyed incorrectly linked the severity of dental decay to concentration of sugars in food. • Most nutritionists were knowledgeable on recommendations including limiting nighttime bottle feeding to reduce childhood caries risk	Knowledge attitudes and practices
Fuller, L.A. (2014) USA [40]	Peer reviewed journal article	To assess the oral health knowledge, confidence and practices of Virginia personnel in the special supplemental food program for Women, Infants and Children (WIC).	Cross-sectional 22-item investigator-designed questionnaire (content validity and reliability established). Some questions sourced from previously tested questionnaires.	159 WIC personnel including -registered dietitians (20%) -nutritionists (33%), −dietetic technicians (5%), −licensed practical nurses (~ 4%) and registered nurses (~ 1%).	• WIC respondents who were over 40 years of age and with 10+ years’ experience were more knowledgeable about caries transmission and the dental decay process than younger and less experienced WIC respondents. • More than half (64%) of respondents were not confident in performing oral screening. • Respondents with higher academic qualifications were more confident (90%) in oral health counselling. • One third of respondents were performing oral screenings for decay. • Most (87%) respondents who were older and more experienced were providing oral health counselling to parents on toothbrushing and were also more likely to provide referrals to dentists.	Knowledge, attitudes and practices
Gold, J.T. (2016) USA [41]	Peer reviewed journal article	Assess the oral health knowledge, practices and attitudes of staff in the Special Supplemental Nutrition Program for Women, Infant and Children (WIC) in north central Florida.	Cross-sectional 28 item questionnaire- developed and validated from previously validated questionnaires.	39 WIC staff including, 9 nutritionists/dietitians.	• Majority of nutritionists (> 78%) were knowledgeable about the importance of maintaining good oral health in children, risk factors and the role of caregivers. • Poor knowledge (33%) around the use of fluoridated toothpaste in children under the age of 2 and nighttime on demand breastfeeding as a risk factor. • All nutritionists were confident in providing oral health counselling for women and children and dental referral if required. • All nutritionists discussed the relationship between dietary choices and caries with parents. • More than two thirds (67%) of nutritionists regularly provided counselling on the importance of tooth brushing and dental visits. • None of the nutritionists were undertaking oral health risk screenings of children and less than half (44%) were referring women and children to dentists.	Knowledge attitudes and practices
Koerber, A. (2006) USA [42]	Peer reviewed journal article	Explore the attitudes and practices of health professionals in a Latino community regarding the association between diabetes and periodontitis to guide interventions for oral health promotion.	Qualitative	Participants (*N* = 14) - 50% Nurses (*n* = 7) - 36% Dentists (*n* = 5) - 14% Nutritionist (*n* = 2) Nutritionist years of experience: ~ 13-20 yrs.	• Knowledge on the relationship between diabetes and its associated symptoms and caries risk was adequate but lacked depth. • All nutritionists considered oral health and diabetes to be important. • Nutritionists were eager to provide oral health education. • None of available resources used by nutritionists discussed the need for oral health and self-care prevention measures with diabetic patients. • Nutritionists were not referring to dentists but demonstrated willingness to do so. • Nutritionists requested more training and suggested development of guidelines and new protocols on the diabetes-periodontitis association as well as patient information handouts and videotapes.	Knowledge attitudes and practices
Shick EA. (2005) USA [43]	Peer reviewed journal article	Examine the effects of knowledge and confidence on dental referral practices among WIC nutritionists in North Carolina.	Cross-sectional 118 item questionnaire- developed from previously tested questionnaires and pilot tested	324 nutritionists	• Most nutritionists were knowledgeable about basic oral hygiene and caries related diet recommendations. • Nutritionists were less aware (33%) of potential caries transmission from carers and importance of fluoride for children. • Nutritionists were very confident about providing oral health counselling and dental referrals (90–96%). They were Less confident (39.3%) about undertaking oral health risk screenings of children. • Confidence in undertaking oral health risk screenings (OR2.12), and making dental referrals (OR 3.02) was associated with more frequent referrals.	Models of care and resources
Karmally W. (2014) USA [44]	Webinar	Provide education on the association between nutrition and oral health conditions and discuss practical strategies for dietitians to integrate oral health information with nutrition counselling.	N/A	Webinar approved for continued professional education	• Webinar discusses the relationship between nutrition, disease and oral health and provides strategies on how dietitians can incorporate oral health into the nutrition assessment (including oral health screening).	Models of care and resources
Brody R.A. (2014) Israel [45]	Peer reviewed journal article	To assess the changes in knowledge and practice of dietitians, working in geriatric care in Israel, following a training program in nutrition focused physical assessment of the oral cavity.	Prospective pilot study using a pre- posttest design. Investigator designed 29 item knowledge pre-test (face and content validity established) and patient data collection forms (practices)	30 dietitians completed the pre-and post-test questionnaires. Testing conducted pre-training, immediately post-training and 12 months post-training (3 timepoints) Training was provided to dietitians which included oral related anatomy, performing extra-oral and intra-oral examination and screening for dysphagia and vitamin deficiencies	• Pretesting suggests that dietitians had limited nutrition focused physical assessment oral health knowledge. At each timepoint following training, knowledge had increased. • Three months post training, dietitians were more likely to perform oral health screenings rather than not assessing or obtaining history from the medical record. • Dietitians were 5.7 x more likely to refer to other allied health professionals 3 months post-training.	Models of care and resources
Jeganathan, S. (2010) Australia [46]	Peer reviewed journal article	Develop and validate a 3-item oral health assessment questionnaire (OHQ) for use by dietitians to screen individuals with HIV at risk of dental complications for referral to dental health services.	Cross-sectional	Tool to be validated: 3 item oral health assessment questionnaire (OHQ) and the Oral health Impact profile 14 (OHIP-14) was administered to 273 clients	• Questionnaire completed by 273 participants. • The OHQ was found to be a valid and sensitive screening tool for dietitians to initiate further investigation for oral health screening and referral to dental professionals. • The sensitivity for the OHQ was 84% and the specificity was 55%. • Compared to the ‘Gold Standard’ Oral health Impact profile-14 (OHIP), the OHQ demonstrated adequate validity (rho = 0.617 (95% CI 0.54, 0.69), *P* < 0.0001).	Models of care and resources
The Albion Centre, oral health promotion working group - NSW Health (2015) Australia [47]	Oral health resource	Oral health screening and referral tool for health professionals (including dietitians) working with individuals with HIV.	N/A	Oral health resource includes three item oral health assessment tool, take home advice for clients, referral pathways and information about common dental problems encountered by individuals with HIV	N/A	Models of care and resources
Pac West MCH - distance Learning Network, University of Washington (2005) USA [48]	Oral health education and screening resource	Provides actions that non-dental and dental health professionals can take to identify individuals at risk of oral health complications and provides guidelines for action.	N/A	Module 5 of an online resource for actions that non-dental health professionals can consider when addressing oral health complications which includes: potential oral health screening and prevention activities, suggestions for screening tools for nutrition related oral health problems, mechanisms for referral and further resources and initiatives	N/A	Models of care and resources
Author (Year)/ Location	Article Type	Aims	Study Design	Details of Study	Conclusion	Focus area
Decker, R.T. (2013) USA [37]	Position statement	To provide the position of The Academy of Nutrition and Dietetics regarding oral health and nutrition as well as describe the roles and responsibilities of dietetics practitioners in oral health education, practice and research.	N/A	Position statement on the role of the dietitians in oral health promotion	• The Academy supports the collaboration and integration of oral health with nutrition services, education and research. • Skills of screening oral health and making referrals is essential for dietitians. • Supports education that reinforces and illustrates the role of nutrition in oral health. • Collaboration between oral health practitioners and dietitians is recommended. • Eating disorders is highlighted as an at-risk group and some oral health manifestations typical to this population, such as xerostomia are specified. However, no specific information exists to detail the support dietitians should provide to this population. • A general table is provided where dietitians can identify oral related manifestations of nutrition deficits.	Guidelines and recommendations
Dietitians Australia and Dental Health Services Victoria (2015) Australia [38]	Joint Position statement	To provide evidence based oral health information to dietitians; to guide how oral health can be incorporated into the various roles of dietitians and to provide a framework for workforce capacity building workforce.	N/A	Joint position statement on the role of the dietitian in oral health promotion	• There is consensus that the dietitian’s role, dependent on setting, should: incorporate oral health screening, especially in priority at risk groups; recognize risk factors; provide nutritional management and/or provide guidance/ referral to oral health professionals; develop and deliver health promotion and education messages. • Specific oral health advice for dietitians is provided for medically compromised/special needs patients including individuals with an eating disorder. • Specific oral health advice for dietitians is provided for medically compromised/special needs patients including individuals with an eating disorder. Key advice includes encouraging good oral hygiene and providing advice on oral care after vomiting.	Guidelines and recommendations
Faine, M.P. (1995) USA [39]	Peer reviewed journal article	Assess the knowledge of the role of diet in dental caries aetiology in nutritionists and dental hygienists.	Cross-sectional	136 registered dietitians and 37 registered dental hygienists	• Awareness varied across different topics related to caries-preventative measures. While 95% of nutritionists recognized fluoride uptake into developing teeth makes dental enamel more resistant to dental caries, only 16% understood that fluoride may hinder bacterial activity. • Most nutritionists (53%) demonstrated awareness of the infectious nature of dental caries but only 38% recognized mothers can transmit bacteria to their children. • Nearly all nutritionists correctly identified high vs. low cariogenic snack foods. Nearly all (97%) of nutritionists were aware of and discussed childhood caries with clients. • Two thirds of nutritionists surveyed incorrectly linked the severity of dental decay to concentration of sugars in food. • Most nutritionists were knowledgeable on recommendations including limiting nighttime bottle feeding to reduce childhood caries risk	Knowledge attitudes and practices
Fuller, L.A. (2014) USA [40]	Peer reviewed journal article	To assess the oral health knowledge, confidence and practices of Virginia personnel in the special supplemental food program for Women, Infants and Children (WIC).	Cross-sectional 22-item investigator-designed questionnaire (content validity and reliability established). Some questions sourced from previously tested questionnaires.	159 WIC personnel including -registered dietitians (20%) -nutritionists (33%), −dietetic technicians (5%), −licensed practical nurses (~ 4%) and registered nurses (~ 1%).	• WIC respondents who were over 40 years of age and with 10+ years’ experience were more knowledgeable about caries transmission and the dental decay process than younger and less experienced WIC respondents. • More than half (64%) of respondents were not confident in performing oral screening. • Respondents with higher academic qualifications were more confident (90%) in oral health counselling. • One third of respondents were performing oral screenings for decay. • Most (87%) respondents who were older and more experienced were providing oral health counselling to parents on toothbrushing and were also more likely to provide referrals to dentists.	Knowledge, attitudes and practices
Gold, J.T. (2016) USA [41]	Peer reviewed journal article	Assess the oral health knowledge, practices and attitudes of staff in the Special Supplemental Nutrition Program for Women, Infant and Children (WIC) in north central Florida.	Cross-sectional 28 item questionnaire- developed and validated from previously validated questionnaires.	39 WIC staff including, 9 nutritionists/dietitians.	• Majority of nutritionists (> 78%) were knowledgeable about the importance of maintaining good oral health in children, risk factors and the role of caregivers. • Poor knowledge (33%) around the use of fluoridated toothpaste in children under the age of 2 and nighttime on demand breastfeeding as a risk factor. • All nutritionists were confident in providing oral health counselling for women and children and dental referral if required. • All nutritionists discussed the relationship between dietary choices and caries with parents. • More than two thirds (67%) of nutritionists regularly provided counselling on the importance of tooth brushing and dental visits. • None of the nutritionists were undertaking oral health risk screenings of children and less than half (44%) were referring women and children to dentists.	Knowledge attitudes and practices
Koerber, A. (2006) USA [42]	Peer reviewed journal article	Explore the attitudes and practices of health professionals in a Latino community regarding the association between diabetes and periodontitis to guide interventions for oral health promotion.	Qualitative	Participants (*N* = 14) - 50% Nurses (*n* = 7) - 36% Dentists (*n* = 5) - 14% Nutritionist (*n* = 2) Nutritionist years of experience: ~ 13-20 yrs.	• Knowledge on the relationship between diabetes and its associated symptoms and caries risk was adequate but lacked depth. • All nutritionists considered oral health and diabetes to be important. • Nutritionists were eager to provide oral health education. • None of available resources used by nutritionists discussed the need for oral health and self-care prevention measures with diabetic patients. • Nutritionists were not referring to dentists but demonstrated willingness to do so. • Nutritionists requested more training and suggested development of guidelines and new protocols on the diabetes-periodontitis association as well as patient information handouts and videotapes.	Knowledge attitudes and practices
Shick EA. (2005) USA [43]	Peer reviewed journal article	Examine the effects of knowledge and confidence on dental referral practices among WIC nutritionists in North Carolina.	Cross-sectional 118 item questionnaire- developed from previously tested questionnaires and pilot tested	324 nutritionists	• Most nutritionists were knowledgeable about basic oral hygiene and caries related diet recommendations. • Nutritionists were less aware (33%) of potential caries transmission from carers and importance of fluoride for children. • Nutritionists were very confident about providing oral health counselling and dental referrals (90–96%). They were Less confident (39.3%) about undertaking oral health risk screenings of children. • Confidence in undertaking oral health risk screenings (OR2.12), and making dental referrals (OR 3.02) was associated with more frequent referrals.	Models of care and resources
Karmally W. (2014) USA [44]	Webinar	Provide education on the association between nutrition and oral health conditions and discuss practical strategies for dietitians to integrate oral health information with nutrition counselling.	N/A	Webinar approved for continued professional education	• Webinar discusses the relationship between nutrition, disease and oral health and provides strategies on how dietitians can incorporate oral health into the nutrition assessment (including oral health screening).	Models of care and resources
Brody R.A. (2014) Israel [45]	Peer reviewed journal article	To assess the changes in knowledge and practice of dietitians, working in geriatric care in Israel, following a training program in nutrition focused physical assessment of the oral cavity.	Prospective pilot study using a pre- posttest design. Investigator designed 29 item knowledge pre-test (face and content validity established) and patient data collection forms (practices)	30 dietitians completed the pre-and post-test questionnaires. Testing conducted pre-training, immediately post-training and 12 months post-training (3 timepoints) Training was provided to dietitians which included oral related anatomy, performing extra-oral and intra-oral examination and screening for dysphagia and vitamin deficiencies	• Pretesting suggests that dietitians had limited nutrition focused physical assessment oral health knowledge. At each timepoint following training, knowledge had increased. • Three months post training, dietitians were more likely to perform oral health screenings rather than not assessing or obtaining history from the medical record. • Dietitians were 5.7 x more likely to refer to other allied health professionals 3 months post-training.	Models of care and resources
Jeganathan, S. (2010) Australia [46]	Peer reviewed journal article	Develop and validate a 3-item oral health assessment questionnaire (OHQ) for use by dietitians to screen individuals with HIV at risk of dental complications for referral to dental health services.	Cross-sectional	Tool to be validated: 3 item oral health assessment questionnaire (OHQ) and the Oral health Impact profile 14 (OHIP-14) was administered to 273 clients	• Questionnaire completed by 273 participants. • The OHQ was found to be a valid and sensitive screening tool for dietitians to initiate further investigation for oral health screening and referral to dental professionals. • The sensitivity for the OHQ was 84% and the specificity was 55%. • Compared to the ‘Gold Standard’ Oral health Impact profile-14 (OHIP), the OHQ demonstrated adequate validity (rho = 0.617 (95% CI 0.54, 0.69), *P* < 0.0001).	Models of care and resources
The Albion Centre, oral health promotion working group - NSW Health (2015) Australia [47]	Oral health resource	Oral health screening and referral tool for health professionals (including dietitians) working with individuals with HIV.	N/A	Oral health resource includes three item oral health assessment tool, take home advice for clients, referral pathways and information about common dental problems encountered by individuals with HIV	N/A	Models of care and resources
Pac West MCH - distance Learning Network, University of Washington (2005) USA [48]	Oral health education and screening resource	Provides actions that non-dental and dental health professionals can take to identify individuals at risk of oral health complications and provides guidelines for action.	N/A	Module 5 of an online resource for actions that non-dental health professionals can consider when addressing oral health complications which includes: potential oral health screening and prevention activities, suggestions for screening tools for nutrition related oral health problems, mechanisms for referral and further resources and initiatives	N/A	Models of care and resources

### Focus area I: guidelines and recommendations on the role of dietitians in oral health

Two position statements on oral health and the role of dietitians were identified: a joint position statement and guideline from the Dietitians Australia (DA, formerly Dietitians Association of Australia) and Dental Health Services Victoria (DSHV) [38], and one from the Academy of Nutrition and Dietetics (USA) [37].

Both position statements supported the belief that nutrition is an integral part of oral health across all life stages and emphasized a shift toward multidisciplinary collaboration for patient-centered care. The identified statements support and stress the need for collaboration between dietitians and dental practitioners for promoting oral health and early intervention in oral health disease [37, 38].

#### Scope of practice

The joint position statement by the DA and DHSV specifically provided advice and areas for intervention which the dietitian can use in addressing oral health risk factors or manifestations. Particularly, potential areas for incorporation of oral health into practice included, during a nutritional assessment, when oral health risk factors can be identified and addressed by providing guidance for management or referral to the oral health practitioner. Additionally, in community or non-acute settings, the dietitian can also participate in OHP [38].

This position statement also highlights priority life stages and risk factors for oral health [38]. In relation to the focus areas of this scoping review, ED and oral health manifestations including dental erosion, mucosal lesions, and altered salivary functions were noted, and key advice was provided to the dietitian on methods to address these issues in practice. Advice included providing education to ‘at risk’ individuals on oral health care after vomiting, appropriate oral hygiene practices and referral to a dental professional where appropriate [38].

Similarly, the position statements from the United States and Australia stated that oral health screening, and referral were part of the dietitian’s role and responsibility in providing comprehensive patient care [37]. The statements highlighted that oral health integration into current dietetic practice can be achieved by the inclusion of oral health screening into the general nutrition assessment with referral as required. The Australian position statement further detailed that oral health screening should be undertaken particularly for at risk populations and various validated oral health screening tools were recommended depending on the setting [50]. As part of the screening process dietitians can recognize oral manifestations of systemic diseases and identify patients at-risk of poor oral health that require referal to dentists. Additionally, the American statement also encouraged the setting of patient care goals with an oral health practitioner [37].

#### Education

Both position statements strongly encouraged oral health education for dietitians. The Australian position statement highlighted that its purpose was to provide a framework for building the confidence and knowledge of current dietitians and for the education of future dietitians in tertiary education [38].

The Academy of Nutrition and Dietetics provided greater detail and outlined their recommendations for interprofessional education in keeping with the recommendations by the Institute of Medicine which calls for the ‘improvement of access to oral health care for vulnerable populations’ [37]. The latter highlighted the integration of didactic and interprofessional education within a curriculum, designed for both dietitians and oral health professionals. This included dietitians in their Bachelor programs receiving lectures and practical tutorials in oral anatomy, physiology and manifestations in disease; clinical experience targeting how to incorporate oral health screening into nutrition assessment; conducting basic nutrition physical assessments with oral and cranial nerve assessment; working with oral health professionals and creating appropriate diets for compromised oral health [37].

### Focus area II: knowledge, attitudes and practices (KAP) of dietitians regarding oral health promotion

An initial search was conducted investigating KAP of dietitians regarding oral health promotion specifically for people with an eating disorder. This search returned no results and hence, a broader search was conducted to include other populations or clinical settings. A total of five articles were identified for this focus area. Due to the variation in the identification of dietitians and nutritionists in the included studies, for simplicity, both dietitians and nutritionists will be collectively referred to as ‘dietitians’ from here forth. One qualitative study focused generally on dietitians oral health knowledge and practice in diabetes management [42], while the remaining four cross sectional studies specifically assessed dietitian KAP in the area of women, infants and children’s (WIC) populations [39–41, 43].

#### Knowledge

Across all the included studies in this focus area dietitians were noted to have mixed awareness of the aetiology, risks and prevention of dental caries [39–43]. Generally, knowledge of the infectious nature of caries varied widely between the cross sectional studies [39, 40, 43]. Particularly, 33 to 97% of dietitians working in the WIC populations were able to identify that mothers can transmit decay causing bacteria to their children [39–41, 43]. The authors did not report why results fluctuated significantly, however, another study did note that dietitians who were older (> 40 years old) and with more years of working experience (> 10 years) were more knowledgeable in this area (87–97%) [40].

Commonly, dietitians had adequate knowledge in their understanding of the impact of dietary choices and behaviors on caries risk [39, 41, 42]. The vast majority (82%) of dietitians involved in WIC population showed consistent awareness of the impact of high risk foods and practices such as night time bottle feeding (82%) and increased risk of dental caries [39] and either knew that periodontal disease could affect glycemic control, or were not surprised by this association [42]. Even so, 66% of dietitians incorrectly identified that the severity of dental caries was linked to the concentration of sugars in food [39].

Awareness of caries prevention strategies was in congruence with the mixed knowledge of caries risks. While one cross sectional study reported that over 95% of dietitians identified strategies for caries prevention such as the use of fluoridated toothpaste [39], others reported that only ‘some’ (33%) staff identified fluoridated toothpaste as a risk minimization strategy [41] and that 34–39% of dietitians were uncertain on the use of fluoride therapies in young children [43]. Furthermore, 60% of dietitians incorrectly identified that children with healthy dentition could make their first dental visit at age three, even though the American Academy of Pediatric Dentistry advises this should occur from as early as one year of age [39]. Finally, 89% of dietitians correctly identified that addressing dental caries in babies was important even though these are not permanent teeth [41].

#### Attitudes

Dietitians had mixed feelings regarding their ability to perform oral health screening. More than half of the dietitians in two cross sectional studies (64–67%) reported not feeling confident in their ability to perform oral screening or identifying tooth decay in children [40, 41]. However, in another cross sectional study, within the same population group, the majority (91%) of dietitians reported they were confident in identifying early childhood caries [43].

In general, dietitian attitudes towards providing OHP education to clients was positive. All dietitians reported feeling confident in their ability to provide education to pregnant women and parents about their child’s oral health [41, 43]. Specifically, dietitians were confident in discussing the role of oral health related dietary habits [40], providing education to families on child dental care, oral health risks during pregnancy and post-partum dental care, dietary and feeding considerations for reducing the risk of dental caries and the need for dental referrals [41, 43].

Additionally, dietitians were noted to be ‘eager to pass this information (OHP) on to patients’ and felt that their clients would find OHP information useful [42]. All dietitians were also confident in referring to dentists where appropriate [41] and were confident (76%) that consumers would take their advice and follow through their referral to dental services [43].

#### Practice

Most dietitians were providing OHP in their practice. Dietitians were generally found to provide counselling on issues such as toothbrushing and fluoride use (70–87%) [39, 40], the role of sugary snacks and drinks in dental decay (67–100%) [39, 40], bottle feeding before bed (100%) [41] and the impact of ‘baby bottle tooth decay’ (97%) [39]. Only 11% of dietitians discussed the role of caries transmission between baby and mother [41]. Although inclusion of OHP in practice was noted to be consistent amongst the included cross sectional studies, dietitians highlighted insufficient access to interpreter services for non-English speaking members, time constraints, inappropriate health insurance cover and resource constraints as common barriers for OHP in clinical practice [42, 43].

Ambivalence of performing oral screening was highlighted in their limited application of the skill in practice. The general consensus was that dietitians did not often perform oral health screening with only up to half of them including these assessments in practice [40, 41]. An even smaller percentage of dietitians (33%) attempted to assess women and caregiver dental health [41].

Interestingly, despite dietitian involvement in basic oral health screening and counselling, there were inconsistencies in the practice of making referrals to dentists. Cross sectional studies reported none to nearly all dietitians (96%) providing referrals for dental care/follow up [41–43]. Dietitians who had more years of working experience (> 10 years) were noted to be more likely to provide referrals [40].

### Focus area III: current models of oral health care and resources for dietitians

Five articles were identified that met focus area III. As sources specific to populations with ED were not identified, the search was broadened to include general and other populations where dietitians are actively involved in oral health care.

#### General population

There is evidence to show that dietitians can play an active role in promoting oral health in the general population. An online continuing professional development training program approved by the Academy of Nutrition and Dietetics in the United States assists dietitians to understand the synergistic relationship between oral health and nutrition, the role of nutrition in the integrity of the oral cavity or progression of oral health related disease, and nutritional counselling [44]. The training specifically outlined nutritional deficiencies/risk and their associated oral manifestations, and oral health considerations through the life stages. Additionally, it also identified the existence of dietetic diagnostic terms for oral health, and encourages proficiency and competency in examining the oral cavity for nutritional deficiencies during screening [44]. An additional training module was identified from the United States on how dietitians can screen for oral health issues, refer to dental health professionals and provide education on nutrition for oral health to the general population [48]. The module also identifies ‘at risk’ populations that would benefit from screening, including populations with an ED, and provides guidance for non-dental health professionals with regard to information required by dental health professionals for making an appropriate and informative referral to dental services.

#### Aged care

The aged care population is an area where dietitians have been shown to play a role in promoting oral health. Brody et al., investigated the impact of a pilot training program for dietitians performing oral nutrition physical assessments in long term residential aged care facilities in Israel [45]. Dietitians were provided a one-and-a-half-day training program on ‘nutrition focused physical assessment’. Dietitians were trained to screen symptoms such as xerostomia (dry mouth), dysgeusia (taste disorder) and pain, extra-oral examination of the face and temporomandibular joint, brief cranial nerve examination, intra-oral examination of mucosa, and signs of micronutrient deficiencies such as lesions. Three to 6 months following training, dietitians were significantly more likely to perform nutrition focused oral physical assessments (*P* < 0.001) and refer to dental health professionals than before training indicating confidence in their ability to perform this role [45].

#### Chronic disease

Dietitians are also a key contributor in identifying People Living with Human Immunodeficiency Virus (PLHIV) who are at risk for poor oral health. A three-item oral health screening tool for dietitians working with this population has been developed and validated in Australia [46]. Jeganathan et al., validated the tool with PLHIV and found it to have high sensitivity (84%), moderate specificity (55%), and a negative predictive value of 77%. Dietitians are currently utilizing this tool in practice in a multidisciplinary health centre for HIV management in New South Wales, Australia [47]. The resource titled ‘Open your mouth’ prompts dietitians and other health professionals to ask clients about their last dental visit, identify the presence of adverse oral health symptoms such as bleeding, provide basic oral health education and a referral pathway for access to dental services [47].

## Discussion

The focus of this scoping review was to identify the role of dietitians in promoting oral health among individuals with an ED by reviewing the evidence and recommendations in this area and any existing oral health models of care and resources available to dietitians.

Australian and American position statements identified that dietitians are in an ideal position to provide OHP. However, importantly, they identified that OHP is in keeping with a dietitian’s scope of practice, and hence, should be, if not already, integrated in standard practice [37, 38]. The scope of practice detailed in these recommendations are limited to providing oral health education, screening and dental referrals to at-risk populations and does not involve diagnosing dental problems which would infringe on the dental profession. This scope of practice is similar to those recommended for non-dental professionals in other settings like antenatal care and aged care [51, 52]. Although these statements were not specific for ED settings, they did highlight vulnerable populations groups at-risk of poor oral health which included individuals with an ED who would benefit from OHP (See Table 1) [38]. In reviews of practice in ED settings, dietitians perform analysis and risk management of nutritional deficiencies and behaviors. However, notably OHP is often not identified as part of this role [32–34]. In fact, a recent review of clinical treatment manuals for adults with an eating disorder worldwide found less than 10 % contained information about dental health [33]. The recognition of ED as a high risk population in these statements is significant, given that ED related oral health issues including impaired dentition, dental sensitivity, and facial muscle wasting [1] would likely affect treatment outcomes, and consequently, impede ED related recovery [33].

While we acknowledge that both position statements make attempts to provide advice regarding how dietitians should implement OHP into practice, the recommendations have limited details pertaining to the ED population (see Table 1) [37, 38]. The paucity of research in this area may have contributed to the lack of clinical practice guidelines which are often developed from high quality evidence [53].

In saying this, although the profession of dietetics has experienced significant growth and development on a professional and global scale since establishment of the Academy of Nutrition and Dietetics in 1917 [54, 55] the role of the dietitian/nutritionist will continue to evolve as the understanding of the scope of practice of dietitians advances [56]. The present lack of OHP guidance for dietitians in these reviews of practice could lead to the postulation that OHP may not currently be seen as a priority or may be considered a novel area of practice. With this in mind, and the scant availability of research showcasing dietetic involvement in oral health, may provide possible insight into why current recommendations for dietetic management of ED fails to include the dietitian’s role in OHP [32–34].

Although no studies that reviewed dietitians providing OHP to individuals with an ED were identified, studies that reviewed OHP across other clinical areas highlighted superficial knowledge of oral health issues, risk factors, and prevention strategies [39–42]. Likely commencing with insufficient oral health education for dietitians at a tertiary level [57] and following through with limited training for professional development [58], it may be conceived that even though these position statements with recommendations are available, they have not translated into dietetic practice. By means of addressing this gap in dietetics, both position statements call for the inclusion of ‘didactic’ education models that demonstrate the role of the dietitian in OHP in tertiary education [37, 38]. This is however not unique to dietitians, but a finding that was shared amongst other non-dental health professionals as a salient barrier to OHP [59–62].

Despite this challenge, some dietitians still felt they were capable of including OHP in their practice [39–43]. This notion of proactivity by dietitians in light of their varying oral health knowledge [39–43] is similar to that shown by other non-dental health professionals. Research reviewing this emerging role in diabetes educators [63], child and family health nurses [61], antenatal care providers including midwives and obstetricians/gynecologists, general practitioners [60, 64], and nursing and carer staff in residential aged care facilities [65, 66] emphasize that non-dental health professionals felt promoting oral health amongst their key population groups was essential in achieving health care goals and part of their scope of practice [60, 61, 63, 64].

The identification of some dietitians already incorporating OHP in their practice was a promising finding. It showcases the skills and capability of dietitians in providing oral health screening, education and referrals to dental services in vulnerable populations [39–42]. However, this should be considered within the context that the general scarcity of evidence could be as a result of clinician hesitance due to inadequate guidelines informing practice, varying confidence in their knowledge and skills on the subject matter, and availability/awareness of referral pathways. The establishment of a model of care can assist in informing implementation into an ED clinical setting and hence, may be considered part of a potential solution [67]. In previous models of care we can see that when dietitians are supported they are capable of delivering OHP to WIC populations [68], children in low income communities [69], an ethnic minority group with diabetes [42] and a population with HIV [46]. Similarly, in studies where child and family health nurses and midwives had a model of care for oral health in practice [61, 64], clinicians confidently and competently were able to include oral health screening and referral in practice.

The most consistent barrier to OHP as identified in this review was the limited availability of professional development materials, both generally and specifically for OHP in ED clinical settings. This is synonymous with other studies involving non-dental health professionals who have also been reported to experience a lack of resources for both the client and health professional [59–62, 64, 66]. Support in the form of training for health professionals in OHP has resulted in improved knowledge and confidence, and clinicians were more likely to incorporate OHP in their practice [64, 70]. Further, these results indicate that involvement by non-dental health professionals allowed for timely intervention and captured ‘at risk’ populations that otherwise may not immediately engage in dental health services due to cost, accessibility, dental anxiety and importantly, the individual’s perception of need for regular dental assessment [24, 71].

In saying this, the development and inclusion of resources for dietitians for OHP in ED clinical settings may not be enough to meet the needs of these health professionals. It is recognized that individuals with a mental health condition may not see oral health as a significant health issue due to more pressing health concerns or due to compromised mental health status [24, 72]. In addition to dietetic input, further consideration of client perceptions to receiving dietitian led oral health promotion and client centered challenges, such as client accessibility and affordability of dental services [22–24], access to appropriate resources and referral pathways for clinicians [73], and the support of dental health professionals in this shared role [37, 38, 73], will all need to be addressed in order for this to be a sustainable and successful early intervention model.

Lastly, it is important to point out that current evidence around the evaluation of oral health models of care involving dietians mainly focus on clinician outcomes in terms of their knowledge, attitude and practices in this area. Curently there is no data to show the benefit of dietitians undertaking oral health promotion on patients’ outcomes nor the acceptability of such interventions by individuals. This is an area that needs further investigation in future studies particularly as capacity building non-dental professionals to promote oral health has been shown to be effective in improving the health outcomes of patients in other settings, including maternal and infant care and aged care [74–76]. For example, the rollout out of a midwifery initiated oral health (MIOH) program in Australia has resulted in a significant improvement in the oral health status and quality of life of pregnant women and was found to be acceptable and feasible to implement into antenatal care practice.

This review is not without limitations. As only articles published in the English language were included, our findings may not be representative of all available literature on this topic. With regard to the included articles, studies were predominantly conducted in the United States, and a modest number of dietitians participated in those studies, limiting the generalizability of results across other countries or regions where the scope of practice and training of dietitians may differ. In light of this novel practice for dietitians, there was no data available on the effectiveness of dietitian initiated oral health promotion on patient outcomes. Further, this review included grey literature which is not peer-reviewed and therefore, the quality of some of the findings may be poor. Nevertheless, due to the limited number of peer reviewed articles on the topic area, the inclusion of grey literature enabled the review to be as exhaustive and comprehensive as it could be.

## Conclusion

Dietitians are in a pivotal position to provide pre-emptive education and screening of oral health and there are examples where dietitians are successfully undertaking this role across various settings. However, this is still an underdeveloped area of dietetics in the ED clinical area. Further research is needed to explore how to support dietitians to promote oral health among the ED populations including any training and screening resources that may enhance their role as well as the effectiveness of these strategies on patient outcomes. It is equally important that future research attempts to understand and evaluate challenges that not only ED patients but any patient seen by a dietitian may encounter in terms of oral health care including their acceptability of dietitian led oral health promotion and accessibility and affordability of dental services.

## Data Availability

not applicable.

## Abbreviations

ED: Eating disorders
DA: Dietitians Australia
DHSV: Dental Health Services Victoria
HIV: Human Immunodeficiency Virus
KAP: Knowledge Attitudes Practices
MESH: Medical Subject Headings
OHP: Oral Health Promotion
PLHIV: People Living with Human Immunodeficiency Virus
WIC: Women infants and children

## References

[1] Aranha A, Eduardo CDP, Cordás TA (2008). Eating disorders. Part I: psychiatric diagnosis and dental implications. J Contemp Dent Pract.

[2] Keski-Rahkonen A, Raevuori A, Hoek HW. Epidemiology of eating disorders: an update. Annual Review of Eating Disorders. Boca Raton: CRC Press; 2018. p. 66–76.

[3] Nielsen S (2001). Epidemiology and mortality of eating disorders. Psychiatr Clin N Am.

[4] McManus S, Meltzer H, Brugha T, Bebbington P, Jenkins R (2014). Adult psychiatric morbidity survey, 2007.

[5] National Eating Disorders Collaboration (2016). Eating Disorders in Australia Canberra: Australians Government, Department of Health.

[6] Hudson JI, Hiripi E, Pope HG, Kessler RC (2007). The prevalence and correlates of eating disorders in the National Comorbidity Survey Replication. Biol Psychiatry.

[7] Le Grange D, Swanson SA, Crow SJ, Merikangas KR (2012). Eating disorder not otherwise specified presentation in the US population. Int J Eat Disord.

[8] Wade TD, Bergin JL, Tiggemann M, Bulik CM, Fairburn CG (2006). Prevalence and long-term course of lifetime eating disorders in an adult Australian twin cohort. Aust N Z J Psychiatry.

[9] Hay P, Girosi F, Mond J (2015). Prevalence and sociodemographic correlates of DSM-5 eating disorders in the Australian population. J Eat Disord.

[10] Australian Institute of Health and Welfare, Welfare AIoHa (2018). Australia's Health 2018.

[11] Hay P, Mitchison D, Collado AEL, González-Chica DA, Stocks N, Touyz S (2017). Burden and health-related quality of life of eating disorders, including avoidant/restrictive food intake disorder (ARFID), in the Australian population. J Eat Disord.

[12] Westmoreland P, Krantz MJ, Mehler PS (2016). Medical complications of anorexia nervosa and bulimia. Am J Med.

[13] Butterfly Foundation. Paying the price: the economic and social impact of eating disorders in Australia. Canberra; 2012. Available from: https://thebutterflyfoundation.org.au/assets/Uploads/Butterfly-report-Paying-the-Price-Executive-Summary.pdf.

[14] Clark DB. Patients with eating disorders: Challenges for the oral health professional. Can J Dent Hyg. 2010;44(4):163–74.

[15] Kantovitz KR, Pascon FM, Rontani RMP, Gaviao MBD, Pascon FM. Obesity and dental caries--A systematic review. Oral Health Prev Dent. 2006;4(2):137–44. 16813143

[16] Hooley M, Skouteris H, Boganin C, Satur J, Kilpatrick N (2012). Body mass index and dental caries in children and adolescents: a systematic review of literature published 2004 to 2011. Syst Rev.

[17] Kisely S, Baghaie H, Lalloo R, Johnson NW (2015). Association between poor oral health and eating disorders: systematic review and meta-analysis. Br J Psychiatry.

[18] Hermont AP, Oliveira PA, Martins CC, Paiva SM, Pordeus IA, Auad SM (2014). Tooth erosion and eating disorders: a systematic review and meta-analysis. PLoS One.

[19] Locker D, Liddell A, Dempster L, Shapiro D (1999). Age of onset of dental anxiety. J Dent Res.

[20] Cash TF, Deagle EA (1997). The nature and extent of body-image disturbances in anorexia nervosa and bulimia nervosa: a meta-analysis. Int J Eat Disord.

[21] Derenne JL, Beresin EV (2006). Body image, media, and eating disorders. Acad Psychiatry.

[22] Wallace B, MacEntee MI (2012). Access to dental care for low-income adults: perceptions of affordability, availability and acceptability. J Community Health.

[23] Wallace B, Browne A, Varcoe C, Ford-Gilboe M, Wathen N, Long P (2015). Self-reported oral health among a community sample of people experiencing social and health inequities: cross-sectional findings from a study to enhance equity in primary healthcare settings. BMJ Open.

[24] Slack-Smith L, Hearn L, Scrine C, Durey A (2017). Barriers and enablers for oral health care for people affected by mental health disorders. Aust Dent J.

[25] Scully CC (2003). Drug effects on salivary glands: dry mouth. Oral Dis.

[26] Marvanova M, Gramith K (2018). Role of antidepressants in the treatment of adults with anorexia nervosa. Ment Health Clin.

[27] Dynesen AW, Gehrt CA, Klinker SE, Christensen LB (2018). Eating disorders: experiences of and attitudes toward oral health and oral health behavior. Eur J Oral Sci.

[28] Silverstein LS, Haggerty C, Sams L, Phillips C, Roberts MW (2019). Impact of an oral health education intervention among a group of patients with eating disorders (anorexia nervosa and bulimia nervosa). J Eat Disord.

[29] Yacoub A, Karmally W. Nutrition in Oral Health. Nutrition in Lifestyle Medicine. Switzerland: Springer; 2017. p. 193–209.

[30] Lifante-Oliva C, López-Jornet P, Camacho-Alonso F, Esteve-Salinas J (2008). Study of oral changes in patients with eating disorders. Int J Dent Hyg.

[31] Bapat S, Asawa K, Bhat N, Tak M, Gupta VV, Chaturvedi P (2016). Assessment of dental nutrition knowledge among nutrition/dietetics students. J Clin Diagn Res.

[32] O’Connor G, Oliver A, Corbett J, Fuller S (2019). Developing clinical guidelines for dietitians treating young people with anorexia nervosa-family focused approach working alongside family therapists. Ann Nutr Disord Ther.

[33] McMaster CM, Wade T, Franklin J, Hart S. A review of treatment manuals for adults with an eating disorder: nutrition content and consistency with current dietetic evidence. Eat Weight Disord Stud Anorexia Bulimia Obes. 2020. p. 1–14. 10.1007/s40519-020-00850-6. 10.1007/s40519-020-00850-6 32002827

[34] McMaster CM, Wade T, Franklin J, Hart S. Development of consensus-based guidelines for outpatient dietetic treatment of eating disorders: a Delphi study. Int J Eat Disord. 2020;53(9):1480–95. 10.1002/eat.23330 32662177

[35] Dietitians Association of Australia (2015). Dietitian or Nutritionist?.

[36] Arksey H, O'Malley L (2005). Scoping studies: towards a methodological framework. Int J Soc Res Methodol.

[37] Touger-Decker R, Mobley C (2013). Position of the academy of nutrition and dietetics: oral health and nutrition. J Acad Nutr Diet.

[38] Dietitians Association of Australia (DAA) DHSVD (2015). Joint position statement on oral health and nutrition.

[39] Faine MP, Oberg D (1995). Survey of dental nutrition knowledge of wig nutritionists and public health dental hygienists. J Am Diet Assoc.

[40] Fuller LA, Stull SC, Darby ML, Tolle SL (2014). Oral health promotion: knowledge, confidence, and practices in preventing early-severe childhood caries of Virginia WIC program personnel. Am Dent Hyg Assoc.

[41] Gold JT, Tomar S (2016). Oral health knowledge and practices of WIC staff at Florida WIC program. J Community Health.

[42] Koerber A, Peters KE, Kaste LM, Lopez E, Noorullah K, Torres I (2006). The views of dentists, nurses and nutritionists on the association between diabetes and periodontal disease: a qualitative study in a Latino community. J Public Health Dent.

[43] Shick EA, Lee JY, Rozier RG (2005). Determinants of dental referral practices among WIC nutritionists in North Carolina. J Public Health Dent.

[44] Karmally W. Nutrition and Oral health: what dietitians should know. Nutri-Bites Webinar Series. Chicago: ConAgra Foods Science Institute; 2014.

[45] Brody RA, Touger-Decker R, Radler DR, Parrott JS, Rachman SE, Trostler N (2014). A novel approach to Oral health assessment training for dietitians in Long-term care settings in Israel: a pilot study of changes in knowledge and practice. Top Clin Nutr.

[46] Jeganathan S, Purnomo J, Houtzager L, Batterham M, Begley K (2010). Development and validation of a three-item questionnaire for dietitians to screen for poor oral health in people living with human immunodeficiency virus and facilitate dental referral. Nutr Diet.

[47] The Albion Centre (2018). Nutrition, Oral Health and HIV.

[48] Pac West MCH distance learning network (2005). Module 5 Screening and referral for nutrition related oral health problems.

[49] Oral Health Promotion Working Group. In: Centre A, editor. Open your mouth: NSW Health; 2013.

[50] Chalmers J, King P, Spencer A, Wright F, Carter K (2005). The oral health assessment tool—validity and reliability. Aust Dent J.

[51] Fricker A, Lewis A (2009). Better oral health in residential care: final report.

[52] Workgroup OHCDPE (2012). Oral health care during pregnancy: a national consensus statement.

[53] Australian Nursing & Midwifery Federation (ANMF) (2014). Definitions of policies, position statements, guidelines, issues papers and fact sheets: Australian Nursing & Midwifery Federation (ANMF).

[54] Academy of Nutrition and Dietetics (2018). Eatright-About us.

[55] Shen X, Tang W, Yu Z, Cai W (2019). The history and development of registered dietitian accreditation systems in China and other comparable countries. Nutr Res.

[56] International Confederation of Dietetic Associations (ICDA) (2016). Dietitian-Nutritionists around the world: Their education and their work.

[57] Scardina G, Messina P. Good oral health and diet. Biomed Res Int. 2012;2012:1–10. 10.1155/2012/720692 PMC3272860 22363174

[58] Johnson L, Boyd L, Rainchuso L, Rothman A, Mayer B (2017). Eating disorder professionals’ perceptions of oral health knowledge. Int J Dent Hyg.

[59] Poudel P, Griffiths R, Wong VW, Arora A, Flack JR, Khoo CL (2020). Perceptions and practices of general practitioners on providing oral health care to people with diabetes-a qualitative study. BMC Fam Pract.

[60] George A, Dahlen HG, Reath J, Ajwani S, Bhole S, Korda A (2016). What do antenatal care providers understand and do about oral health care during pregnancy: a cross-sectional survey in New South Wales, Australia. BMC Pregnancy Childbirth.

[61] Veale M, Ajwani S, Johnson M, Nash L, Patterson T, George A (2016). The early childhood oral health program: a qualitative study of the perceptions of child and family health nurses in South Western Sydney, Australia. BMC Oral Health.

[62] Barnett T, Hoang H, Stuart J, Crocombe L, Bell E (2014). Utilisation of oral health services provided by non-dental health practitioners in developed countries: a review of the literature. Community Dent Health.

[63] Poudel P, Griffiths R, Wong VW, Arora A, Flack JR, Khoo CL (2018). Perceptions and practices of diabetes educators in providing oral health care: a qualitative study. Diabetes Educ.

[64] Dahlen HG, Johnson M, Hoolsema J, Norrie TP, Ajwani S, Blinkhorn A (2019). Process evaluation of the midwifery initiated oral health-dental service program: perceptions of midwives in greater Western Sydney, Australia. Women Birth.

[65] Hoad-Reddick G (1991). A study to determine oral health needs of institutionalised elderly patients by non dental health care workers. Community Dent Oral Epidemiol.

[66] Chalmers JM, Pearson A (2005). A systematic review of oral health assessment by nurses and carers for residents with dementia in residential care facilities. Special Care Dent.

[67] Agency for Clinical Innovation (ACI). A practical guide on how to develop a Model of Care at the Agency for Clinical Innovation. Chatswood; 2013.

[68] Taylor E, Marino D, Thacker S, DiMarco M, Huff M, Biordi D (2014). Expanding oral health preventative services for young children: a successful interprofessional model. J Allied Health.

[69] Biordi DL, Heitzer M, Mundy E, DiMarco M, Thacker S, Taylor E (2015). Improving access and provision of preventive oral health care for very young, poor, and low-income children through a new interdisciplinary partnership. Am J Public Health.

[70] Heilbrunn-Lang AY, De Silva AM, Lang G, George A, Ridge A, Johnson M (2015). Midwives’ perspectives of their ability to promote the oral health of pregnant women in Victoria, Australia. BMC Pregnancy Childbirth.

[71] Freeman R (1999). The psychology of dental patient care: barriers to accessing dental care: patient factor. Br Dent J.

[72] BSDH Bsfdaoh (2000). Oral health care for people with mental health problems: guidelines and recommendations.

[73] Marshall R, Spencer A (2006). Accessing oral health care in Australia.

[74] Nyongesa NN. Implementing an evidence-based oral health assessment tool (OHAT) in a nursing home. North Dakota: North Dakota State University; 2013.

[75] Maher L, Phelan C, Lawrence G, Dawson A, Torvaldsen S, Wright C (2012). The early childhood Oral health program: promoting prevention and timely intervention of early childhood caries in NSW through shared care. Health Promot J Aust.

[76] George A, Sousa MS, Kong AC, Blinkhorn A, Norrie TP, Foster J (2019). Effectiveness of preventive dental programs offered to mothers by non-dental professionals to control early childhood dental caries: a review. BMC Oral Health.

